# Supplementary material for: Association Analysis of Maximum Standardized Uptake Values Based on 18F-FDG PET/CT and EGFR Mutation Status in Lung Adenocarcinoma
Source: J Pers Med. 2023 Feb 23;13(3):396. doi: 10.3390/jpm13030396 (PMC10058931; doi:10.3390/jpm13030396)
Supplement: Supplementary file 1 [file jpm-13-00396-s001.zip › jpm-2171818-supplementary.pdf]

Table S1. Comparison of the characteristics of different smoking history groups.

Smoking history	Negative	Positive	P-value
N	235	131	
Age (years)	63.8 (9.5)	64.6 (8.6)	0.416
Gender			<0.001
Female	192 (81.7%)	2 (1.5%)	
Male	43 (18.3%)	129 (98.5%)	
Nodule type			<0.001
Solid	130 (55.3%)	106 (80.9%)	
Subsolid	105 (44.7%)	25 (19.1%)	
Location			0.947
Upper right	74 (31.5%)	45 (34.5%)	
Middle right	12 (5.1%)	8 (6.1%)	
Lower right	46 (19.6%)	26 (19.9%)	
Upper left	64 (27.2%)	33 (25.2%)	
Lower left	39 (16.6%)	19 (14.5%)	
Shape			0.454
Round/oval	125 (53.2%)	75 (57.3%)	
Polygon/Irregular	110 (46.8%)	56 (42.8%)	
Lobulation sign	195 (83.0%)	115 (87.8%)	0.221
Burr sign	123 (52.3%)	76 (58.0%)	0.296
Bronchial sign	144 (61.3%)	55 (42.0%)	<0.001
Vacuolation sign	34 (14.5%)	17 (13.0%)	0.693
Pleural indentation sign	163 (69.4%)	74 (56.5%)	0.013
Vascular bundle sign	148 (63.0%)	68 (51.9%)	0.039
Tumor long axis (mm)	25.40 (20.15-35.00)	32.00 (19.90-47.65)	0.003
CEA (ng/ml)	3.08 (1.50-11.05)	5.56 (3.00-16.28)	<0.001
SUV_{max}	8.87 (3.20-14.86)	13.88 (8.09-19.41)	<0.001
Clinical stage			<0.001
I	136 (57.9%)	39 (29.8%)	
II	5 (2.1%)	9 (6.9%)	
III	31 (13.2%)	28 (21.4%)	
IV	63 (26.8%)	55 (42.0%)	
EGFR			<0.001
Wild-type	60 (25.5%)	78 (59.5%)	
Mutant	175 (74.5%)	53 (40.5%)	

Note: Mean (SD) / Median (Q1-Q3) / N (%).
